# Narratives to revert overconsumption: human-nature interdependence and Circular Economy

**DOI:** 10.1186/s40100-023-00259-6

**Published:** 2023-06-14

**Authors:** M. Borrello, L. Cembalo, V. D’Amico

**Affiliations:** 1 Department of Agricultural Sciences, AgEcon and Policy Group, University of Naples Federico II - Via Università 100, 80055 Portici, Italy; 2 Center of Plant Sciences, Scuola Superiore Sant’Anna, Piazza Dei Martiri Della Libertà 33, 56127 Pisa, Italy

**Keywords:** Sustainability transition, Sustainable consumption, Sustainable lifestyle, Affluence, Sufficiency, Frugality, Coupled human and natural systems

## Abstract

Policy and practitioners’ initiatives to stimulate sustainable consumption have so far failed to have notable impact on individuals’ behaviors. The current commentary is a plea to social and sustainability scientists, particularly to economists dealing with sustainable agri-food systems, to dig deeper into the notion of narratives to trigger societal dynamics that stir consumers toward more sufficient lifestyles. As dominant cultural narratives have a critical role in shaping shared meanings and acceptable behaviors, in the future they could guide dramatic changes in individuals’ conduct, triggering drastic modifications of current consumption patterns. Based on the power that concepts as the Circular Economy and the Anthropocene have had in the recent past, a future step to develop an ecological worldview across society, and nourish individual identities deeply committed with the preservation of natural ecosystems, is working on narratives based on the notion of human-nature interdependence*.*

## Background

The world is living an unprecedented and remarkable upheaval. The economic recession due to the COVID-19 pandemic and the energy crisis produced by the Russo-Ukrainian war have shown the fragility of global industries and supply chains. Besides rocking the assumption of limitless economic growth, these shocks are bringing out our dependence on constant and reliable flows of products and services, and the dramatic consequences of sudden interruptions of their supply. However, the socio-technical interpretation of current events may lead to consider this crisis as a window of opportunity (see Geels 2019) to nurture a “new normal”, by questioning the taken-for-granted social practices related to consumption (Borrello et al. 2022a). Taking the agri-food system as an example, the urgency of food system transformation seems to be irrefutable (Webb et al. 2020), including people reconnection to the ecological dimension of food and civic engagement to build resilient agri-food futures (Sage 2014). Accordingly, even the World Economic Forum has highlighted the urgent need to reflect and reimagine our world and to implement structural changes in our consumption behavior (see the *Great Reset* initiative https://www.weforum.org/great-reset/).

Advocating this viewpoint and based on the recent *Scientists’ warning on affluence* (Wiedmann et al. 2020), this commentary addresses the issue of how designing long-term desirable changes in sustainable consumption habits, particularly reducing overconsumption. According to Wiedmann and colleagues (*ibid.*, p.3), “Since the level of consumption determines total impacts, affluence needs to be addressed by reducing consumption, not just greening it”. It is no accident that a burgeoning area of scientific literature advocates a viewpoint for which sustainability goals should be pursued targeting a shift toward sufficiency, which focus on reducing absolute demand by redirecting consumption choices toward what is “enough” to satisfy human needs (Gorge et al. 2015; Bocken & Short 2016; Spangenberg & Lorek 2019; Brand-Correa et al. 2020). This would need a dramatic change in consumer culture, in which overconsumption patterns are no longer normalized throughout society, nor perceived as instrumental for wellbeing and for a satisfactory life (Borrello et al. 2022a).

However, while the search of low impact technical solutions to produce consumable goods seems to take its course and progress, what needs to be done to make sufficient lifestyles more acceptable is far from being settled. Contrariwise, the dominant eco-modernist paradigm does not challenge high-consumption habits, but assumes that, to live sustainably, people keep buying more things, such as electric cars, recyclable coffee pods, energy efficient appliances, to name some. Furthermore, the lack of emphasis devoted to reverting overconsumption dynamics is accompanied by inadequate long-term strategies able to effectively address people behavioral changes. Initiatives promoting sustainable consumption strongly rely on an attitude-based approach: individuals who have internalized a set of environmentalist values are considered more likely to act sustainably. However, evidence on the value-behavior gap (Brunsø et al. 2004) shows that environmental engagement is not growing in accordance with the increase in global environmental concern. In the agri-food domain, only a minor share of individuals embraces sustainable food consumption practices; and eating less meat (Willett et al. 2019), consuming less energy and water, and wasting less food (Foden et al. 2019) still represent unresolved issues at household level. This suggests the need of a renewed endeavor at the foundation of the inquiry about sustainable consumption, to support and integrate strategies adopted so far by policy makers and practitioners.

## The role of narratives

Drawing from this argument, we make a plea to social and sustainability scientists to dig deeper into the notion of *narratives* and their role to trigger environmentally sustainable societal dynamics (Chabay et al. 2019). Narrative theory posits that human beings adopt available dominant narratives to organize the interpretation of everyday experiences and, therefore, to guide their behavior. As navigating the complexity of our surrounding world is highly demanding in terms of cognitive effort, narratives provide their users with coherent stories, in which life events are rendered intelligible by their systematical relations. Narratives perform three essential functions: “1. They structure, prioritize, and ascribe meaning to experiences and beliefs. […] 2. They provide orientation for facing uncertain and unfamiliar contexts […] 3. They facilitate sense making and decision making in highly complex social–ecological systems …” (*ibid.*, p.5). These functions give people the opportunity to shape their identity and social personhood in line with a clear and organized cultural landscape, thus offering guidelines on how one should behave in each situation. By this logic, the relevance of effective sustainability narratives to change consumption behavior is that they would prepare people to make everyday choices in harmony with a certain “sustainability script”: doing differently would essentially violate their sense of being.

While recently the term “narrative” has entered international food policy agendas (HLPE 2020), scientific contribution investigating narratives of food consumption are isolated. However, some authors have shown the complexity of social, cultural, and material factors shaping narratives surrounding food consumption (Paddock 2017a; 2017b; Borrello and Cembalo, 2020). As narratives take on such critical role, the Global Sustainability Strategy Forum (https://www.iass-potsdam.de/en/research/global-sustainability-strategy-forum)—a group of active sustainability experts—has recently recommended scientist, business, politics, and civil society to engage in the co-creation of narratives able to actually induce transformations toward sustainability.

One of the most pressing issues on this topic is identifying visionary narratives able to act as catalyst of sustainable individual behaviors, and even revert overconsumption. If the engagement in narratives co-creation started, identifying—and then generating—conditions conducive of transformative social change is not straightforward. What visionary narrative can effectively act as catalyst of sustainable individual behaviors, and even revert overconsumption? This commentary seeks to contribute to this debate making a plea for the development of narratives evoking human-nature interdependence.

### Nature-inspired narratives to revert overconsumption

In line with Milkoreit and colleagues (2017), we posit that what is missing in current sustainability narratives, in the process of meaning-making, and in collective visions of the future, is the very idea of nature. According to Sheperd (1982), the city child finds Manhattan more comprehensible than a forest; and the Italian novelist Italo Calvino (1966) once wrote: “… children, who are thoroughly urban, seem to misunderstand any references to natural things”. This is the consequence of a worldview considering nature only as a cornucopia indefinitely exploitable by humans. Working against this state of things, the concept of “Anthropocene” has recently contributed to recall that, in the real world, humanity is part of nature (see Marcus et al. 2010), introducing a powerful narrative on human–environment relationships that recognizes human agency at the interface with the Earth system (Leichenko and O’Brien, 2021). Sizing through a geological conceptual tool the magnitude of human intervention’s impacts on nature has strong emotional resonances for many people. Based on this, we call for a joint effort in shaping narratives able to stimulate the interpretation of the world in terms of *Coupled Human And Natural Systems* (CHANS) (Liu et al. 2021). CHANS science adopts a comprehensive approach to incorporate the interconnections and dynamics between human and natural systems, both within and across different scales. It emphasizes the interactions and feedbacks between the human and natural components within these systems. This integrated framework is essential for comprehending the complexity of the Anthropocene era and generating creative solutions to the unparalleled global challenges we face. Albeit CHANS identifies an integrated framework to understand the complexity of society-nature interactions, it can also have inspirational power to feed narratives able to profoundly reshape individuals’ interpretation of nature. Such narratives would lead people to understand and interiorize the existence and relevance of society-nature interdependence and coevolution, with the ultimate goal to spread a more ecological worldview (Du Plessis & Brandon 2015). This would imply reconceptualizing humans as members of the community of life, thus adapting humans’ intentions and actions to the functioning of the biosphere and realizing strong supportive symbiotic relationships with other living systems. For example, Luederitz et al. (2017) considers the narrative behind citizen groups willing to create ecologically advanced and socially integrated urban communities (*e.g.*, the Vauban neighborhood in Freiburg., Germany). These groups share “visionary imaginaries of an ecological-oriented system framing which emphasizes environmental ethics, natural conservation and technology use to facilitate simple living” (*ibid.*, p.399). Therefore, their *ecotopian solutions* aim to transitioning “toward greater social–ecological integrity through creating living spaces outside of conventional, state-led governance and support of narrative aligned belief systems and practices” (*ibid.*, p.398).

### Circular Economy as nature-inspired narrative

Following this line of reasoning, we highlight the need to subject the current influential Circular Economy (CE) narrative to a new scrutiny. With more than 18,000 records on Scopus and an annual average growth rate of published articles of about 33% in the last 10 years, CE has become the vehicle of meaning and purpose of much of present sustainability research effort. Some authors have criticized the CE concept for being vague and unorganized (Korhonen et al. 2018). CE has also been blamed for neglecting thermodynamic limits to recycling and the reduction in expected gains due to the increased efficiency of resource use, *i.e.,* microeconomic responses such as the rebound effect (Georgescu-Roegen 1971; Berkhout et al. 2000; Skene, 2018). However, the CE concept has channeled decades of scientific research on industrial ecosystems and regenerative design outside academia, attracting business interest and providing politics with a course of action for sustainability work (see, for example, the Circular Economy Action Plan of the European Green Deal). Also, the *circle metaphor* is an immediate signifier particularly relevant for social sense making, that makes CE the more likely candidate to replace the notion of “sustainable development” (Genovese & Pansera 2021). Given the magnitude of its potential impact on socially constructed meanings, it is a wonderment that the CE narrative has become prominent without a political agenda that critically addresses overconsumption. Several years after its appearance, the CE discourse remains detached from a serious ideological reflection on individual behaviors and lifestyles, leaving unquestioned current overconsumption practices. As a result, the CE transformative ambition has been downplayed, leaving space for a refurbished, technocentric and weak version of sustainability, where citizens are still identified with passive individuals called to consume green product, recycle, and adhere to service centered business models (Lombardi & Cembalo 2022). We claim that this eco-modernist stance improperly makes overconsumption unproblematic and that the CE momentum deserves that individual behavioral aspects are reprioritized. For the sake of a thoughtful consideration of planetary boundaries, the circle metaphor should diverge from suggesting only technological silver bullets. Instead, it should rehabilitate its nature-inspired archetypical meaning—that calls to rethink our understanding of connections between humans and nature—turning it into a tenet of a sustainability narrative able to inspire deeply engaged social identities.

## Implications for agri-food systems

Agri-food systems are remarkably fit to convey such kind of CE and human-nature interdependence narratives. Røpke (2009, p. 2495) proposes an ecological perspective of consumption in which “human society can be seen as a metabolic organism appropriating resources from the environment, transforming them for purposes useful for humans, and finally discarding them as waste”. When this process is linear, CE suggests closing the loop through “materials metabolisms” that replicate ecological processes (Cembalo et al. 2020). Food falls in the domain of biological metabolisms, where biological nutrients flow from one company to another, to consumers and back, mimicking the processes of organic mineralization-synthesis occurring in biological ecosystems. In this scenario, consumers should be aware that responsible use of resources in the domestic sphere and recycling are their contribution to maintaining the ecological processes supporting food production. To this aim, social and sustainability scientists interested in agri-food systems, particularly agricultural and food economists, are called to integrating more complex approaches to consumers’ behavior and to deepen their understanding of human relationships with the natural world. Economist can give crucial contribution to food systems transformation regarding the consumption sphere, with microeconomic analysis shedding light on consumers’ choices under scarcity and individuals’ trade-offs between different objectives (Di Vita et al. 2020; Fan 2021; Borrello et al. 2022b). However, as dominant narratives have the power to shape, reproduce, and transform scientific paradigms (Paschen & Ison 2014), economists’ contribution to challenge overconsumption and foster sufficient lifestyles has been negligible to date. On one hand, the prevailing eco-modernist narrative makes agricultural and food economists mainly focused on strategies to increase the consumption of environmentally sustainable products, whose production impacts are often only slightly lower than their conventional counterparts (Montero-Navarro et al. 2021). On the other hand, agricultural and food economists are hardly engaged in observing, understanding, and co-creating with consumers narratives, as well as seeking to transfer these into education and policy making agendas as powerful elements for sense making. Given this state of things, a legitimate self-reflection for agricultural and food economists could inspire fruitful collaborations with other social sciences and environmental humanities, meant to draw attention to more complex and nuanced understandings of social systems and related food consumption behaviors. By combining diverse theoretical approaches, interdependencies, feedback loops, and complex dynamics between economic systems, social behavior, and environmental outcomes can be identified (see, for example, Borrello et al., 2020c). Engaging also various stakeholders—including policymakers, communities, and industry representatives—in the development of shared conceptual repertoires, the resulting narratives can better reflect the real-world complexities and be more relevant to decision-makers (see, for example, Galafassi et al. 2018). Furthermore, by linking economic modeling, cost–benefit analysis, and econometric techniques with insights from qualitative research methods, participatory approaches, and systems thinking, this interdisciplinary blending can result in a more comprehensive understanding of sustainability issues (Pluye & Hong 2014). This interdisciplinary collaboration—encompassing different perspectives on human behavior, methodologies, and value considerations—can help to developing narratives that go beyond purely economic efficiency and account for broader societal and environmental values.

## Conclusions

The following scheme summarizes the content of this commentary. As behavioral approaches seem to have failed to date the goal to revert overconsumption, visionary sustainability narratives reconceptualizing humans as part of nature are needed. These narratives should guide interpretation and agency across complex socio-ecological systems, giving cultural and emotional resonance to individuals’ choices harming or preserving the environment. Interiorizing human-nature interdependence and coevolution in a coherent collection of assumptions to think about the world is the way toward an ecological worldview shaping decisions across society. This worldview would de-legitimize and de-normalize unsustainable consumption choices, potentially leading to more sufficient lifestyles.

**Figure:**
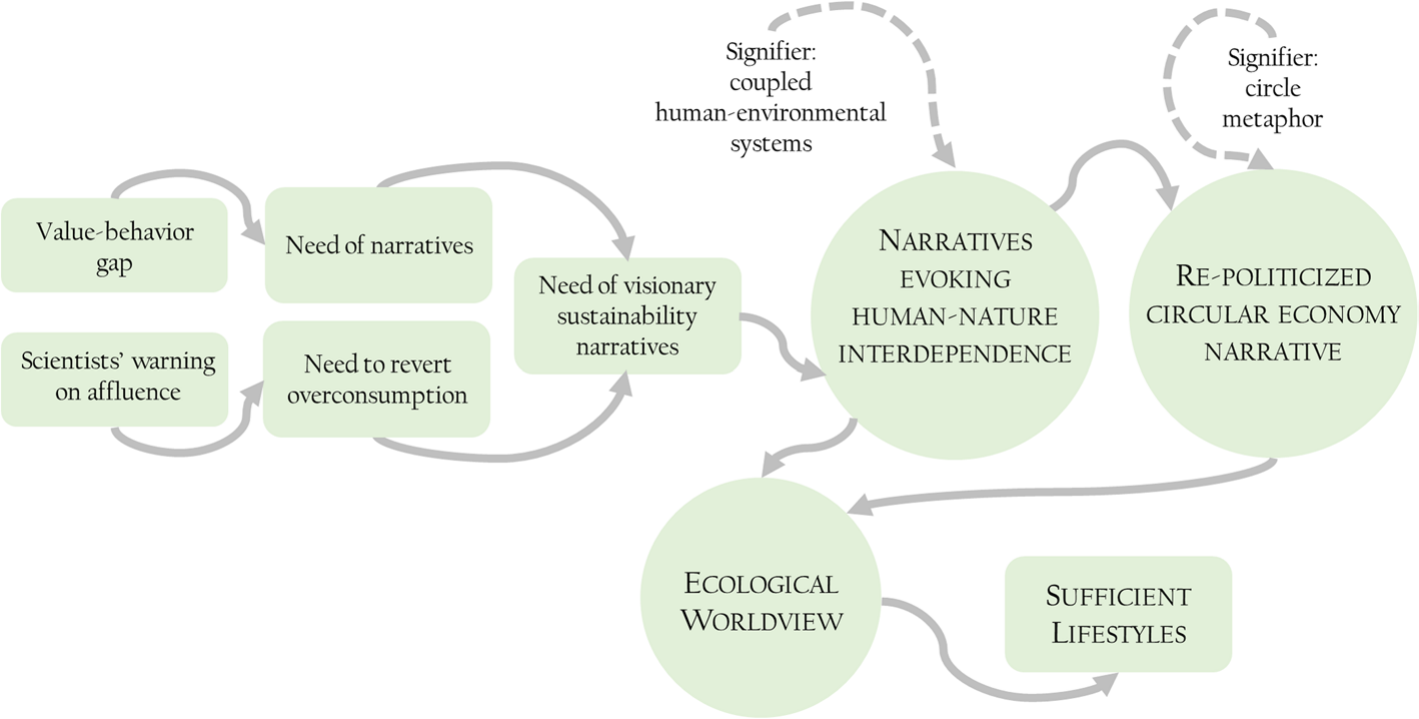
Human-nature interdependence and Circular Economy as visionary sustainability narratives to foster sufficient lifestyles—Conceptual model

The socio-ecological challenges of the twenty-first century have found us unprepared. Current hegemonic sustainability discourses promise technical, organizational, and managerial solutions, optimistically assuming them as a panacea to deal with natural resource scarcity while driving economic growth in the next decades. More realistically, long-term sustainability goals will be achieved through dramatic changes in individuals’ behavior, entailing drastic modifications of current consumption patterns. A narrative approach may be the pathway to pursue such ambitious transformations. Based on the power that concepts as the Circular Economy and the Anthropocene have had in the recent past, a future step for sustainability scientists is working on narratives revolving around the notion of human-nature interdependence. The ambition is to nourish social identities deeply committed with the preservation of natural ecosystems, for which sufficient consumption may be the new normal.

## Data Availability

Not applicable.

## Abbreviations

CE: Circular Economy
CHANS: Coupled human and natural systems
